# The impact of extra-valvular cardiac damage on mid-term clinical outcome following transcatheter aortic valve replacement in patients with severe aortic stenosis

**DOI:** 10.3389/fcvm.2022.1039208

**Published:** 2022-12-01

**Authors:** Costanza Pellegrini, Charlotte Duesmann, Tobias Rheude, Amelie Berg, Hector A. Alvarez-Covarrubias, Teresa Trenkwalder, N. Patrick Mayr, Friederike Schürmann, Philipp Nicol, Erion Xhepa, Michael Joner

**Affiliations:** ^1^Klinik für Herz- und Kreislauferkrankungen, Deutsches Herzzentrum München, Technical University Munich, Munich, Germany; ^2^Institut für Anästhesiologie, Deutsches Herzzentrum München, Technical University Munich, Munich, Germany; ^3^Deutsches Zentrum für Herz- und Kreislauf-Forschung (DZHK) e.V. (German Center for Cardiovascular Research), Partner Site Munich Heart Alliance, Munich, Germany

**Keywords:** aortic stenosis, cardiac damage, femoral, transcatheter aortic valve implantation (TAVI), transthoracic echocardiogram, stages of cardiac damage

## Abstract

**Aims:**

To quantify extra-valvular cardiac damage associated with severe aortic valve stenosis (AS), a novel staging model was proposed. This study aimed to validate this model in patients undergoing transcatheter aortic valve replacement (TAVR) as well as to assess its prognostic impact.

**Methods and results:**

Based on echocardiographic findings, the following stages were applied: isolated AS (stage 0), left ventricular (LV) damage (stage 1), left atrial or mitral valve damage (stage 2), pulmonary hypertension or tricuspid regurgitation (stage 3), or right ventricular dysfunction (stage 4). The primary endpoint was 2-year all-cause mortality. The distribution across stages was 0.8% at stage 0, 7.5% at stage 1, 63.3% at stage 2, 18.3% at stage 3, and 10.1% at stage 4. All-cause mortality increased at all stages 1–4 (12.1%, 18.2%, 26.6%, and 28.2%; *p* = 0.023). In the multivariate model, the stage of cardiac damage, age, New York Heart Association (NYHA) class III/IV, peripheral artery disease, and previous pacemaker were independent predictors of the primary endpoint.

**Conclusions:**

Patients treated for severe AS show a high prevalence of extra-valvular cardiac damage. An increase in stage is associated with higher 2-year all-cause mortality. The application of this staging model may add value to current treatment algorithms.

## Introduction

Aortic valve stenosis (AS) is the most commonly acquired heart valve defect in industrial nations, and its incidence rate is 4.4%/year in patients above 65 years of age (1). Surgical or transcatheter aortic valve replacement (TAVR) remains the standard therapy for severe AS. Currently, an indication for the treatment of AS is driven by the severity of stenosis and the clinical manifestation of symptoms. The decision to recommend conventional surgical vs. catheter-based aortic valve replacement depends on patients‘ age and comorbidities (2). With the exception of a reduced left ventricular (LV) ejection fraction (< 50%), no additional structural cardiac changes are currently included in the decision algorithm, although extra-valvular cardiac damage is believed to occur over time. After an initial adaptive response with concentric hypertrophy to maintain cardiac output in severe AS, this initially beneficial mechanism leads to both systolic and diastolic LV dysfunction, the time point at which most patients will develop symptoms (3). The damage then extends to the left atrium due to elevated filling pressures and leads to left atrial dilatation and remodeling with an increased risk of atrial fibrillation and mitral regurgitation (4). A secondary increase in pulmonary artery pressure may eventually lead to tricuspid regurgitation and right ventricular dysfunction (5, 6). Although concomitant cardiac damage has been described in numerous studies in patients with severe AS (7–10) and is expected to affect long-term outcomes, it is currently under debate whether it can be used as a prognostic parameter and thus be relevant for decision-making regarding the optimal timing of therapy. Recently, in the PARTNER II trial, Généreux et al. proposed the quantification of extra-valvular cardiac damage in patients with AS and found that this classification was significantly correlated with a worse outcome when applied to patients undergoing TAVR for severe AS (11). Therefore, we aimed to validate extra-valvular cardiac damage staging initially proposed by Généreux et al. in patients undergoing TAVR for severe AS stenosis in a real world, single-center population and to assess its impact on mid-term outcome. To increase statistical power, we dichotomized the proposed cardiac damage stages corresponding to isolated left heart dysfunction (0–2) vs. stages 3–4, i.e., the damage extending to the pulmonary circulation and right heart.

## Methods

### Patient population

Between 2011 and 2016, 1,118 patients underwent TAVR for symptomatic severe AS at the Deutsches Herzzentrum München, Munich, Germany. For the scope of this analysis, only patients treated by transfemoral access and without previous valve surgery (aortic, mitral, or tricuspid) were considered (*n* = 1,063). Of these, for 841 (79%) patients a complete echocardiographic data set was obtained at baseline to allow staging. For a detailed study flow chart, see Supplementary Figure S1. All cases were discussed in the multidisciplinary heart team, and a consensus on the therapeutic strategy was achieved. All patients gave their written informed consent for the procedure. TAVR was performed in a hybrid operating suite under general anesthesia or conscious sedation. Data were prospectively collected and classified according to the Valve Academic Research Consortium criteria (12). Data collection was approved by the ethics committee of the Faculty of Medicine at Technische Universität München.

### Echocardiography and staging

Echocardiography was performed prior to the procedure in the hospital's echocardiography department. Two-dimensional, color-wave, pulsed-wave, and continuous Doppler technique images were obtained in parasternal and apical views according to current recommendations (13). The following parameters were assessed: LV ejection fraction, LV mass index, E/e', left atrial volume, aortic valve area, mean and maximum transaortic valve gradient, the grade of aortic, mitral, and tricuspid regurgitation, pulmonary artery pressure, and right ventricular function.

The extent of extra-aortic cardiac damage was retrospectively categorized into five stages according to the model previously described by Généreux et al. (11): stage 0: showing no extra-valvular cardiac damage; stage 1 with isolated LV damage defined by increased LV mass, LV ejection fraction < 50% or E/e'>14; stage 2 with left atrial dilatation >34 ml/m^2^, moderate-to-severe mitral regurgitation or the presence of atrial fibrillation; stage 3 with pulmonary hypertension ≥ 60 mmHg or moderate-to-severe tricuspid regurgitation; and stage 4 with moderate-to-severe right ventricular dysfunction.

### Follow-up and definition of endpoints

Follow-up data were prospectively collected during routine ambulatory visits at the outpatient clinic, by referring to the treating physician, hospital documentation, or through telephone interview. Events were censored after 2 years or when the last event-free contact was made.

The primary endpoint of this analysis was to assess the impact of extra-valvular cardiac damage on all-cause mortality, while the secondary endpoint comprised a composite of all-cause mortality and rehospitalization for worsening congestive heart failure (CHF).

### Statistical analysis

Categorical and ordinal variables are expressed as frequencies and proportions and were compared using the chi-squared or Fisher exact test. Continuous variables are expressed as mean with standard deviation (SD) or median with interquartile range and compared using the Student‘s *t*-test or the Mann–Whitney *U*-test, as appropriate. Survival and event rates during follow-up were calculated as Kaplan–Meier estimates, and a comparison of cumulative event rates between these groups was performed by a log-rank test. Two-sided *p* < 0.05 was considered statistically significant for all analyses. To increase statistical power, cardiac damage stages were dichotomized in stages 0–2 (isolated left heart dysfunction) vs. stages 3–4 (left and right heart dysfunction). To identify significant predictors of the primary and secondary endpoints, univariate Cox regression analysis was performed for all variables; clinically relevant variables yielding *p* < 0.05 in the univariate analysis were used for the multivariate Cox regression analysis.

IBM SPSS Statistics (Version 28, SPSS, Inc. Chicago, IL, USA) and R (Version 1.4.1103, The R Foundation, Vienna, Austria) were used for analyses.

## Results

### Patient population and cardiac damage staging

Overall, of the 841 patients enrolled in this study, the mean age was 81.1 ± 5.8 years and 47.3% (398/841) were women with a median logistic EuroScore of 14.2% (8.97–22.06). Pre-procedural echocardiographic staging classified 0.8% (7/841) of patients having no cardiac damage (stage 0), and 7.5% (63/841) and 63.3% (532/841) were graded in stages 1 and 2, respectively. 18.3% (154/841) of patients were in stage 3, while 10.1% (85/841) were in stage 4. The single component of each stage is depicted in Supplementary Table S1. Baseline characteristics according to the stage of cardiac damage are shown in Table 1. The number of patients suffering from New York Heart Association (NYHA) functional class III/IV increased stepwise with each stage of cardiac damage (*p* for the trend < 0.001). Furthermore, the logistic EuroScore showed a non-linear increase with each stage of cardiac damage, and patients in stages 2–4 had more frequently undergone coronary artery bypass grafting and previous pacemaker implantation.

**Table 1:** Baseline characteristics according to stages of cardiac damage.

	**Stage 0**	**Stage I**	**Stage II**	**Stage III**	**Stage IV**	***p* for the trend**
	***n* = 7**	***n* = 63**	***n* = 532**	***n* = 154**	***n* = 85**	
Age (years)	79.9 ± 4.0	79.1 ± 5.7	81.1 ± 5.7	82.2 ± 6.2	81.0 ± 5.9	**0.010**
Females	4 (57.1)	27 (42.9)	247 (46.4)	87 (56.5)	33 (38.8)	0.072
Body mass index (kg/m^2^)	25.4 ± 3.2	26.8 ± 4.6	27.0 ± 4.9	25.7 ± 4.4	26.7 ± 4.5	**0.037**
EuroScore I (%)	9.0 [6.0–12.6]	8.5 [6.2–13.8]	12.8 [8.7–18.0]	23.1 [13.5–31.2]	20.9 [13.5–35.3]	**< 0.001**
NYHA class III/IV	2 (28.6)	32 (50.8)	346 (65.0)	118 (76.6)	70 (82.4)	**< 0.001**
Arterial hypertension	7 (100.0)	55 (87.3)	491 (92.3)	141 (91.6)	70 (82.4)	**0.047**
Diabetes mellitus	1 (14.3)	23 (36.5)	143 (26.9)	40 (26.0)	33 (38.8)	0.082
COPD	1 (14.3)	7 (11.1)	77 (14.5)	34 (22.1)	14 (16.5)	0.175
Coronary artery disease	5 (79.4)	50 (79.4)	386 (72.6)	109 (70.8)	67 (78.8)	0.529
Percutaneous coronary intervention	4 (57.1)	26 (41.3)	231 (43.4)	73 (47.4)	43 (50.6)	0.604
Coronary artery bypass graft	0 (0)	3 (4.8)	46 (8.6)	11 (7.1)	23 (27.1)	**< 0.001**
Peripheral artery disease	0 (0)	5 (7.9)	67 (12.6)	15 (9.7)	15 (17.6)	0.252
Stroke (minor or major)	1 (14.3)	7 (11.1)	60 (11.3)	16 (10.4)	10 (11.8)	0.995
Previous Pacemaker	0 (0)	1 (1.6)	61 (11.5)	24 (15.6)	19 (22.4)	**0.002**
Previous dialysis	0 (0)	0 (0)	5 (0.9)	4 (2.6)	0 (0)	0.272

Data are expressed as *n* (%), mean ± standard deviation or median with [interquartile ranges].

COPD, Chronic pulmonary obstructive disease; NYHA, New York Heart Association functional class.

Table 2 shows baseline characteristics according to the dichotomized stages of cardiac damage. Patients in stages 3–4 were ~1 year older than patients in stages 0–2 (81.8 ± 6.1 vs. 80.8 ± 5.7 years; *p* = 0.033), and presented with significantly higher rates of comorbidities including higher rates of chronic obstructive pulmonary disease, previous coronary artery bypass grafting, and previous pacemaker implantation. Overall, patients in stages 3–4 had a higher preoperative logistic EuroScore I [22.9% (13.5–32.7) vs. 12.2% (8.1–17.6); *p* < 0.001] and higher rates of NYHA classes III–IV (78.7 vs. 63.1%; *p* < 0.001).

**Table 2:** Baseline characteristics according to dichotomized stages of cardiac damage.

	**Stage 0-II**	**Stage III-IV**	***p*-value**
	***n* = 602**	***n* = 239**	
Age (years)	80.8 ± 5.7	81.8 ± 6.1	0.033
Females	278 (46.2)	120 (50.2)	0.291
Body mass index (kg/m^2^)	27.0 ± 4.8	26.0 ± 4.4	0.007
EuroScore I (%)	12.2 [8.1–17.6]	22.9 [13.5–32.7]	< 0.001
New York Heart Association class III/IV	380 (63.1)	188 (78.7)	< 0.001
Arterial hypertension	553 (91.9)	211 (88.3)	0.105
Diabetes mellitus	167 (27.7)	73 (30.5)	0.417
Chronic pulmonary obstructive disease	85 (14.1)	48 (20.1)	0.033
Coronary artery disease	441 (73.3)	176 (73.6)	0.909
Percutaneous coronary intervention	261 (43.4)	116 (48.5)	0.173
Coronary artery bypass graft	49 (8.1)	34 (14.2)	0.008
Peripheral artery disease	72 (12.0)	30 (12.6)	0.812
Stroke (minor or major)	68 (11.3)	26 (10.9)	0.863
Previous Pacemaker	62 (10.3)	43 (18.0)	0.002
Previous dialysis	5 (0.8)	4 (1.7)	0.283
Mean transvalvular gradient (mmHg)	46.2 ± 15.3	38.2 ± 14.8	< 0.001
Aortic valve area (cm^2^)	0.72 ± 0.19	0.69 ± 0.20	0.012

Data are expressed as *n* (%), mean ± standard deviation or median with [interquartile ranges]. *p* values < 0.05 are highlighted bold.

### Two-year outcome according to cardiac damage

**Figure 1:**
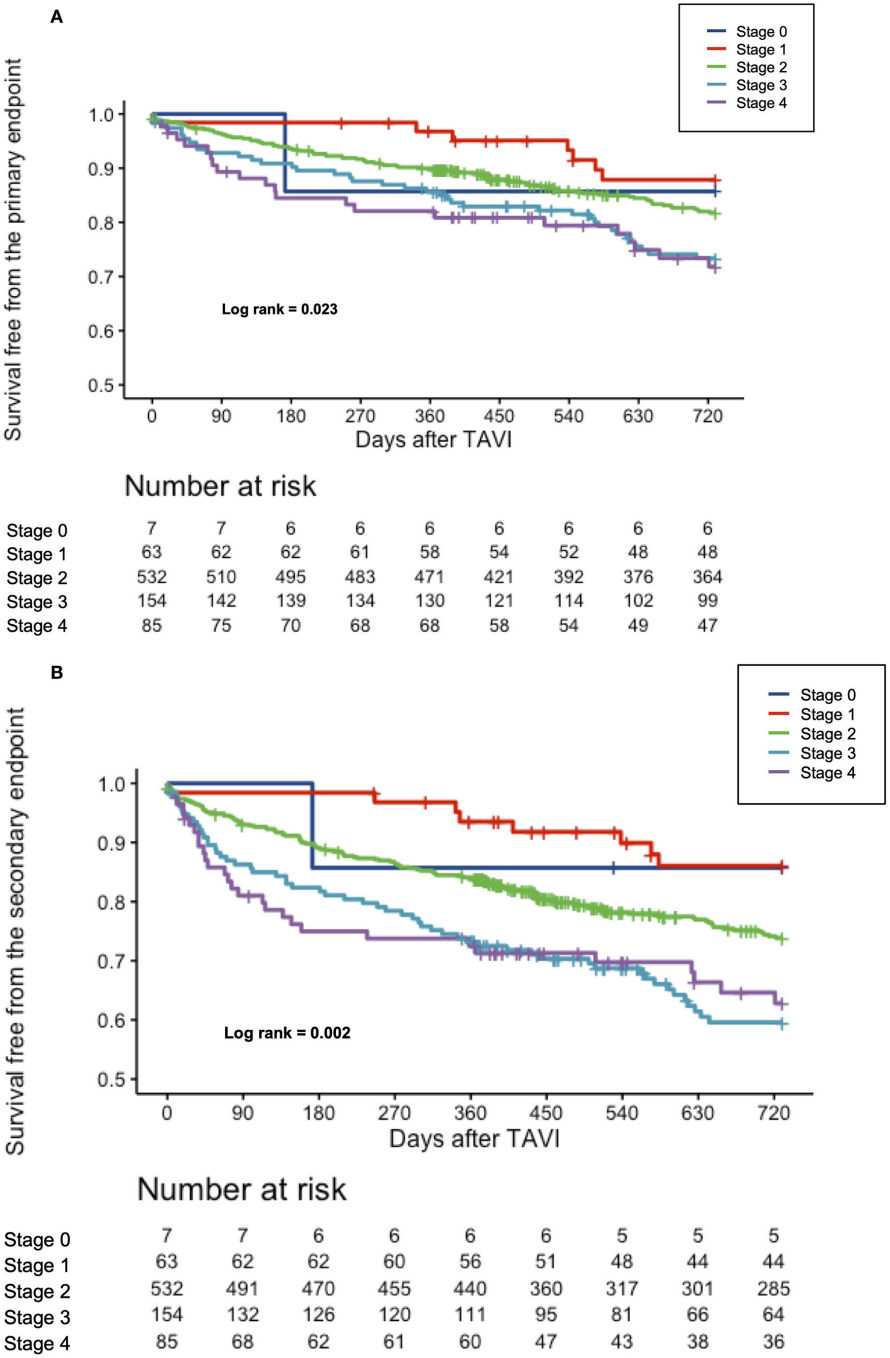
**(A)** Cumulative incidence of all-cause mortality according to the stages of cardiac damage. Kaplan–Meier curves showing cumulative incidence of all-cause mortality according to the stages of cardiac damage. **(B)** Cumulative incidence of the composite all-cause mortality or rehospitalization for worsening CHF according to the stages of cardiac damage. Kaplan–Meier curves showing cumulative incidence of the composite all-cause mortality according to the stages of cardiac damage.

The median follow-up was 851 days (438–1,700), and a 2-year follow-up was completed for 722/841 (85.9) patients. At 2 years, all-cause mortality in the overall population was 20.3%, while the composite of all-cause mortality and CHF occurred in 26.5%. Clinical outcomes at 2 years differed between the individual stages of cardiac damage, as depicted in Table 3. The Kaplan–Meier survival curves showed a significant stepwise increase in all-cause mortality and the composite endpoint of all-cause mortality or rehospitalization for CHF across all stages of cardiac damage, except for stage 0 due to small numbers (Figures 1A,B).

**Table 3:** Two-year outcome according to stage of cardiac damage.

	**Stage 0**	**Stage I**	**Stage II**	**Stage III**	**Stage IV**	***p*-value**
	***n* = 7**	***n* = 63**	***n* = =532**	***n* = 154**	***n* = 85**	
All-cause mortality	1 (14.3)	7 (12.1)	91 (18.2)	39 (26.6)	22 (28.2)	0.023
CHF	0 (0)	3 (5.2)	53 (11.2)	34 (25.7)	14 (18.6)	< 0.001
All-cause mortality or rehospitalization for CHF	1 (14.3)	8 (14.0)	128 (26.1)	57 (40.4)	29 (37.2)	< 0.001

Data are expressed as *n* (%).

CHF, hospitalization for congestive heart failure.

**Figure 2:**
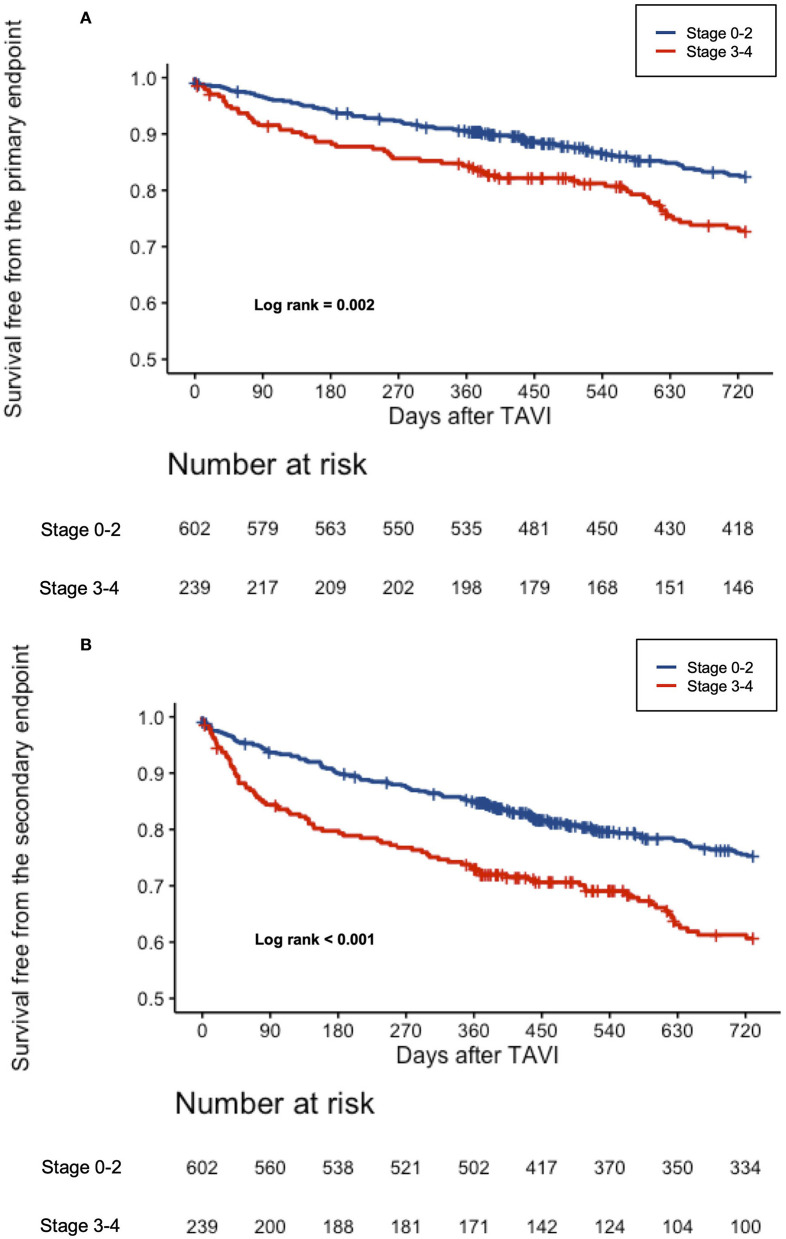
**(A)** Cumulative incidence of all-cause mortality according to the dichotomized stages of cardiac damage. Kaplan–Meier curves showing cumulative incidence of all-cause mortality according to the dichotomized stages of cardiac damage. **(B)** Cumulative incidence of the composite all-cause mortality or rehospitalization for worsening CHF according to the dichotomized stages of cardiac damage. Kaplan–Meier curves showing cumulative incidence of the composite all-cause mortality according to the dichotomized stages of cardiac damage.

The results were even more pronounced using the dichotomized stages of cardiac damage (stages 0–2 vs. 3–4), where the Kaplan–Meier survival analysis paralleled prior findings with almost doubled event rates for the primary and secondary endpoints for stages 3–4 of cardiac damage (Figures 2A,B). Supplementary Table S2 displays a 2-year clinical outcome according to the dichotomized stages of cardiac damage.

### Prognostic relevance of cardiac damage

To assess the impact of cardiac damage on the outcome, univariate and multivariate Cox regression analyses for the primary and secondary endpoints were performed; significant results are shown in Table 4. The stage of cardiac damage, age, NYHA class III/IV, peripheral artery disease, and previous pacemaker implantation were significant predictors of all-cause mortality. In detail, each step increase in the stage of cardiac damage resulted in a 30% higher risk for 2-year all-cause mortality (odds ratio (OR) 1.306, 95% confidence interval (CI): (1.051–2.622); *p* = 0.016).

**Table 4:** Univariate and multivariate Cox proportional hazard analysis for variables yielding p < 0.05 for all-cause mortality and the composite of all-cause mortality or rehospitalization for worsening CHF at 2 years.

	**Univariate analysis**		**Multivariate analysis**	
	**Hazard ratio (95% CI)**	***p*-value**	**Hazard ratio (95% CI)**	***p*-value**
**All-cause mortality**
Stage	1.434 (1.165–1.764)	0.001	1.306 (1.051–1.622)	0.016
Age	1.055 (1.022–1.089)	0.001	1.045 (1.013–1.079)	0.006
NYHA III/IV	2.089 (1.350–3.233)	0.001	1.746 (1.116–2.732)	0.015
Peripheral artery disease	1.615 (1.021–2.556)	0.041	1.636 (1.033–2.591)	0.036
Previous Pacemaker	1.9511.276–2.981	0.002	1.649 (1.075–2.532)	0.022
**All-cause mortality or rehospitalization for worsening CHF**
Stage	1.468 (1.244–1.731)	< 0.001	1.331 (1.119–1.584)	0.001
Age	1.054 (1.028–1.081)	< 0.001	1.048 (1.021–1.076)	< 0.001
NYHA III/IV	2.253 (1.582–3.207)	< 0.001	1.824 (1.268–2.624)	0.001
COPD	1.440 (1.022–2.030)	0.037	1.411 (0.993–2.005)	0.055
Peripheral artery disease	1.515 (1.038–2.211)	0.031	1.523 (1.042–2.225)	0.030
Previous Pacemaker	1.940 (1.376–2.733)	< 0.001	1.660 (1.175–2.346)	0.004

CHF, hospitalization for congestive heart failure; COPD, chronic obstructive pulmonary disease; NYHA, New York Heart Association functional class.

Similar findings were found for the secondary endpoint, where the stage of cardiac damage, age, NYHA III/IV, peripheral artery disease, and previous pacemaker were significant predictors in multivariate analysis. In particular, each increase in the stage of cardiac damage resulted in a 33% higher risk for all-cause mortality or rehospitalization for CHF [OR 1.331, 95% CI: (1.119–1.584); *p* = 0.001].

## Discussion

The main findings of this study can be summarized as follows: in a large, single-center, real-world population, the application of the staging classification proposed by Généreux et al. showed a high prevalence of significant extra-valvular cardiac damage among patients referred for TAVR with symptomatic severe AS. Furthermore, clinical outcome worsened stepwise with increasing stage of cardiac damage. In multivariate analyses, increased cardiac damage stage independently predicted adverse outcome in terms of all-cause mortality and a composite of all-cause mortality or rehospitalization for worsening CHF. This finding was even more pronounced when considering a simplified dichotomized staging classification of stages 0–2 vs. 3–4.

In the original study of 1,661 patients with a mean age of 73 years undergoing aortic valve replacement for severe AS, Généreux et al. found the stage of cardiac damage to be the strongest predictor of 1-year all-cause mortality along with frailty and oxygen-dependent chronic obstructive pulmonary disease. For each increase in the stage of cardiac damage, the 1-year mortality risk increased by 45%. Similarly, in a strong population of 1,189 patients with severe AS with a mean age of 73 years, Vollema et al. confirmed the negative impact of cardiac damage staging on the outcome, in addition to age, previous myocardial infarction, renal function, and surgical or transcatheter AVR, which were all independently associated with all-cause mortality in the multivariable analysis (14). The current analysis differs from previous studies in that it only recruits patients undergoing TAVR, leading to obvious demographic differences, with a mean age of 81 years. Nevertheless, we found that cardiac damage staging was an independent predictor of all-cause mortality up to 2 years after TAVR, confirming its negative impact and reproducibility across different study populations. Similarly, Okuno et al. found that elderly patients with a high prevalence of right ventricular damage had a 5- to 7-fold increased risk of mortality at 1 year (15). It is not surprising that in this elderly population, age and clinical manifestation with regard to NYHA class III/IV were also independently associated with mortality. Recently, the cardiac damage staging model was applied to patients with asymptomatic moderate-to-severe AS, and 61% of patients were found to be in stage ≥2, with a markedly increased risk of mortality during a follow-up. The staging system may be applied to optimize the timing of TAVR procedures and identify asymptomatic patients with severe AS who may benefit from early valve replacement (16). It should also be noted that mortality and worsening of CHF in the current TAVI population, which is older and has many comorbidities, are multifactorial events that are not solely influenced by the extent of cardiac damage. Indeed, the presence of peripheral artery disease was associated with a poorer 2-year clinical outcome. Further, COPD, which was previously was associated with increased mortality after TAVR (17, 18), showed a trend toward a poorer clinical outcome, albeit without reaching statistical significance. To achieve the optimal clinical outcome, operators need to consider such comorbidities when planning treatment.

An indication for treatment is currently based on the extent of valve stenosis severity, symptoms, and LV ejection fraction, and guidelines further consider the presence of additional risk factors, such as STS-score or EuroScore, frailty, or other major organ dysfunction (19). The ongoing extension of an indication with TAVR will likely entail the need to tailor treatment and identify those who benefit the most. Finally, establishing the simplification of the staging classification using the dichotomized stages presented in this paper and differentiating patients with isolated LV damage from those with damage extending to the pulmonary circuit and right ventricle may help to increase the efficiency and accuracy of the clinical decision process in identifying patients who may best benefit from TAVR. Similarly, a recent retrospective analysis identified structural alterations in left and right heart morphology as sensitive indicators of poor prognosis after TAVR by applying unsupervised machine learning (20).

Furthermore, the question arises as to the best timing of treatment: symptomatic manifestation of AS is the principle underlying decision-making to proceed to TAVR; however, the onset of symptoms is often delayed and may manifest when irreversible damage has already occurred. In the analysis by Généreux et al., the stage of cardiac damage was a stronger predictor of mortality compared to any hemodynamic parameter, suggesting that the impact of extra-valvular damage secondary to AS often persists even after successful aortic valve replacement (11). In these cases, patients may possibly benefit from early intervention when irreparable extra-valvular damage has not yet occurred, especially since there is evidence that LV (21) and RV dysfunction can improve immediately after the relief of LV obstruction with TAVR (22). The treatment of this specific subset of patients is still controversial: while guidelines recommend treatment for asymptomatic very severe AS or rapid progression with low surgical risk, evidence is scarce (23, 24). Meanwhile, recent evidence suggests that early surgery in asymptomatic patients with aortic stenosis reduces mortality and death from cardiovascular causes (25), so further research on the optimal timing of transcatheter replacement is crucial. The ongoing randomized clinical Evaluation of TAVR Compared to SurveilLance for Patients With AsYmptomatic Severe Aortic Stenosis (Early TAVR) trial (ClinicalTrials.gov Identifier: NCT03042104) will soon shed light on this debate by enrolling asymptomatic patients with severe AS and comparing clinical surveillance to TAVR.

## Strengths and limitations

Despite the inclusion of 841 strong, real-world patients treated at a high-volume center and presenting complete data with a 2-year follow-up, this analysis has some limitations: first of all, it is an observational study with self-adjudication of events, lacking a central core laboratory. Furthermore, intra- and inter-observer variability inherent in echocardiography cannot be ruled out as a possible confounder in this analysis. There was no systematic assessment of symptomatic status improvement during the follow-up.

## Conclusions

Patients undergoing TAVR for severe AS show a high prevalence of extra-valvular cardiac damage. An increase in the stage of cardiac damage is associated with higher 2-year all-cause mortality. The application of this staging model may add value to current treatment algorithms. Further prospective studies are warranted to confirm the additive value of the proposed staging system in patients with severe AS.

## Impacts on daily practice

Severe AS may be associated with extra-valvular cardiac damage. Recently, Généreux et al. proposed a staging model with stages 0–4 according to the severity of the echocardiographic findings of extra-valvular cardiac damage. In a large population of patients undergoing TAVR, we found a high prevalence of extended cardiac damage. Further, the stage of cardiac damage was associated with higher 2-year all-cause mortality and was found to be an independent predictor of 2-year all-cause mortality in a multivariable model. The application of this staging model may add value to current treatment algorithms for patients with severe AS undergoing TAVR.

## Data availability statement

The original contributions presented in the study are included in the article/Supplementary material, further inquiries can be directed to the corresponding author/s.

## Ethics statement

The studies involving human participants were reviewed and approved by Ethics Committee of the Faculty of Medicine at Technische Universität München. The patients/participants provided their written informed consent to participate in this study.

## Author contributions

CP, CD, TR, AB, PN, FS, and HA-C contributed to the conception and implementation of the study, to acquire and process the data, perform the statistical analysis, and to write the manuscript. TT, NM, and HA-C verified the analytical methods. EX and MJ supervised the analysis and writing of the manuscript. All authors discussed the results and contributed to the final manuscript.

## Conflict of interest

The authors declare that the research was conducted in the absence of any commercial or financial relationships that could be construed as a potential conflict of interest.

## Publisher's note

All claims expressed in this article are solely those of the authors and do not necessarily represent those of their affiliated organizations, or those of the publisher, the editors and the reviewers. Any product that may be evaluated in this article, or claim that may be made by its manufacturer, is not guaranteed or endorsed by the publisher.

## Supplementary material

Supplementary Figure 1 Study flow chart.

Supplementary Table 1 Incidence of cardiac damage stages and their individual components.

Supplementary Table 2 2-year outcome according to dichotomized stages of cardiac damage.

## Abbreviations

AS: aortic valve stenosis
CHF: congestive heart failure
LV: left ventricular
TAVR: transcatheter aortic valve replacement.
