# Supplementary material for: The impact of extra-valvular cardiac damage on mid-term clinical outcome following transcatheter aortic valve replacement in patients with severe aortic stenosis
Source: Front Cardiovasc Med. 2022 Dec 1;9:1039208. doi: 10.3389/fcvm.2022.1039208 (PMC9751869; doi:10.3389/fcvm.2022.1039208)
Supplement: Supplementary Figure 1 — Study flow chart. [file Data_Sheet_1.docx]

**Supplemental Material**

**Supplemental Table 1. Incidence of cardiac damage stages and their individual components**

| Stage 0 | 7/841 (0.8) |
| --- | --- |
| Stage I | 63 (7.5) |
| Stage II | 532 (63.3) |
| Stage III | 154 (18.3) |
| Stage IV | 85 (10.1) |
| **Individual components of cardiac damage types among the study population** | |
| **Stage I** | 740/841(88.0) |
| Increased LV Mass Index | 694/830 (83.6) |
| E/e´>14 | 491/750 (65.4) |
| LV Ejection fraction <50% | 233/841 (27.7) |
| **Stage II** | 758/841(90.1) |
| Indexed left atrial volume >34mL/m2 | 622/729 (85.3) |
| Moderate-Severe mitral regurgitation | 191/841 (22.8) |
| Atrial fibrillation | 395/841 (47.0) |
| **Stage III** | 190/841 (22.6) |
| Pulmonary hypertension >60mmHg | 115/841 (13.7) |
| Moderate-Severe tricuspid regurgitation | 119/841 (14.2) |
| **Stage IV** | 85/841 (10.1) |
| Moderate-severe right ventricular dysfunction | 85/841 (10.1) |

**Supplemental Table 2. 2-year outcome according to dichotomized stages of cardiac damage**

|  | Stage 0-II  n=602 | Stage III-IV  n=239 | p-value |
| --- | --- | --- | --- |
| All-cause mortality | 99 (17.5) | 61 (27.2) | 0.002 |
| CHF | 56 (10.4) | 48 (23.4) | <0.001 |
| All-cause mortality or rehospitalization for CHF | 137 (24.7) | 86 (39.3) | <0.001 |

Data are expressed as n (%)

Abbreviations: CHF, hospitalization for congestive heart failure.

**
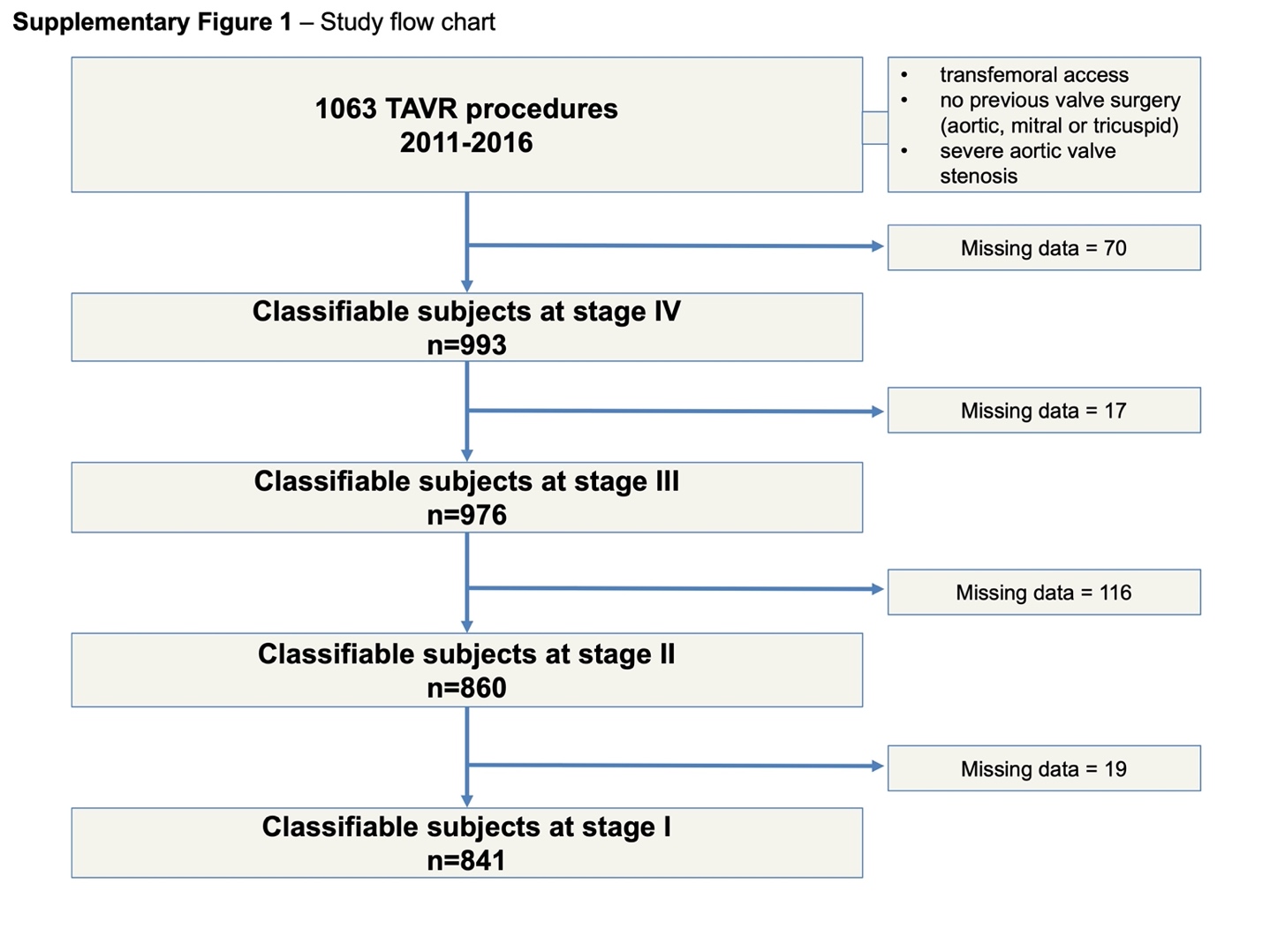
**
